# Effect of degumming degree on the structure and tensile properties of RSF/RSS composite films prepared by one-step extraction

**DOI:** 10.1038/s41598-023-33844-2

**Published:** 2023-04-24

**Authors:** Meng Li, Wei Tian, Yangxiao Yu, Yao Zhang, Boyu Zhang, Jianmei Xu, Jiannan Wang

**Affiliations:** College of Textile and Clothing Engineering, Soochow University, No. 199 Ren-Ai Road, Suzhou Industrial Park, Suzhou, 215123 Jiangsu China

**Keywords:** Materials science, Biomaterials, Soft materials, Structural materials

## Abstract

Regenerated silk fibroin (RSF) and regenerated sericin (RSS) have attracted much attention for tissue engineering due to excellent biocompatibility and controllable degradation. However, pure RSF films prepared by existing methods are brittle, which limits applications in the field of high-strength and/or flexible tissues (e.g. cornea, periosteum and dura). A series of RSF/RSS composite films were developed from solutions prepared by dissolving silks with different degumming rates. The molecular conformation, crystalline structure and tensile properties of the films and the effect of sericin content on the structure and properties were investigated. Fourier transform infrared spectroscopy (FTIR) and X-ray diffraction results revealed more β-sheets in films prepared by boiling water degumming than in Na_2_CO_3_-degummed RSF_C_ film. Analysis of mechanical properties showed that the breaking strength (3.56 MPa) and elongation (50.51%) of boiling water-degummed RSF/RSS film were significantly increased compared with RSF_C_ film (2.60 MPa and 32.31%), and the flexibility of films could be further improved by appropriately reducing the degumming rate.

## Introduction

Silk is a natural protein fibre comprising silk fibroin (70–80 wt%) and glue-like sericin (20–30 wt%)^1^. In recent decades, regenerated silk fibroin (RSF) has established itself as an indispensable biopolymer for tissue engineering applications due to its satisfactory cytocompatibility, adjustable biodegradability and low immunogenicity^2^.

Silk fibroin can be processed into films, nanofibres, particles, hydrogels and porous scaffolds for tissue engineering applications. However, different tissue repair protocols require scaffold materials with different mechanical properties. For example, materials used for cornea repair should possess good wettability and permeability, and suitable strength and flexibility to comply with eye movement^3^. For periosteum tissue engineering, materials should not only possess remarkable elasticity and ductility, but also be tough enough to resist stress stimulation during tissue growth^4^. However, pure RSF films reported to data are brittle with poor flexibility, which limits their application in dura, periosteum and cornea tissue engineering. In order to enhance the performance of pure RSF films, other substances were introduced for reinforcement in the preparation process, such as graphene oxide, hydroxyapatite or formamide^5–7^. However, the flexibility of RSF-based films remains to be further improved.

Molecular weight (or degree of polymerisation) is a key factor determining the mechanical properties of polymer materials^8^. The molecular weight of RSF mainly depends on the degumming and dissolving methods used to prepare silk fibres. In terms of degumming, Na_2_CO_3_, urea and various proteases are the most commonly used degumming agents. Studies indicate that Na_2_CO_3_ degumming can damage the hierarchical structure of silk fibres and accelerate their dissolution in solvents, resulting in decreased molecular weight^9^. The molecular weight of RSF can be > 100 kDa when degummed by urea and protease^10,11^. In terms of dissolving methods, RSF solutions can be obtained by dissolving silk fibres in lithium bromide (LiBr), hexafluoroisopropanol, CaCl_2_/H_2_O/EtOH ternary solvent or formic acid. The molecular weights of RSF solutions prepared by Na_2_CO_3_ degumming and dissolving in the above reagents were 179 ± 4, 176 ± 3, 132 ± 4 and 128 ± 5 kDa, respectively^12^. Aznar-Cervantes et al*.* reported that the elongation and breaking strength of RSF nanofibres were significantly affected by different dissolving methods^13^. These studies reconfirmed that the molecular weight of RSF was mainly determined by the extraction method, and this in turn affected the mechanical properties of RSF materials.

Sericin is a globular protein with high viscosity that can protect core fibres and is rich in hydrophilic groups^14^. In general, sericin is usually discarded as waste in the preparation of RSF materials, leading to environmental pollution^15^. Regenerated sericin (RSS) also possesses good biocompatibility, but its use in biomaterials has not been explored due to concerns over immunogenicity, but this was recently addressed^16^. Accordingly, RSS has been explored for use in wound dressing, cornea repair, bone/cartilage repair and drug delivery^17–20^.

The molecular weight of sericin also varies depending on the extraction method. The molecular weight of RSS collected by Na_2_CO_3_ degumming was significantly lower than that obtained by boiling water degumming^21,22^. In addition, a study indicated that silk fibroin fibres can be destroyed by the complete removal of sericin during degumming^23^, inevitably reducing the molecular weight of RSF.

We developed a boiling water degumming method to obtain RSF/RSS composite solutions by controlling the degumming rates in order to retain high-molecular-weight silk protein^24^. This method was not necessary to prepare RSF and RSS separately and then blend them, hence we considered it a one-step extraction method. Subsequently, a series of RSF/RSS composite films were prepared by polyethylene glycol diglycidyl ether (PEG-DE) crosslinking^25^ and the structures and tensile properties of the films were investigated to explore their application prospects for tissue engineering. The present work provides a new method to obtain silk protein films with improved flexibility using fewer chemicals and without loss of sericin, benefitting environmental protection.

## Materials and methods

### Materials

*Bombyx mori* raw silk (20/22D, 6A) was purchased from Hai An Tian Xin Silk Industry (Nantong, China). Dialysis bag (14 kDa, MD44) was purchased from Union Carbide (Danbury, CT, USA). Sodium carbonate (Na_2_CO_3_, ≥ 99%) and PEG-DE were purchased from Sinopharm Chemical Reagent Co. Ltd. (Shanghai, China). Lithium bromide (LiBr, ≥ 99%) was purchased from Finecollection Institute of Chemical Industry (Hefei, China).

### Degumming and dissolving of silk

*B. mori* raw silk was treated in boiling distilled water at a bath ratio of 1:50 (w/v) for 1, 3 or 5 h, and boiling distilled water was replaced every hour. Na_2_CO_3_ (0.06 wt%) degumming served as a control^26^. After drying, silks with various degree of degumming (Table 1) were dissolved in 9.3 M LiBr at 65 °C for 1 h, then dialysed against distilled water at 4 °C for 72 h. Finally, silk protein aqueous solutions were concentrated to 50 mg/mL.

**Table 1:** Degumming method and degumming rate of silk. Results are means ± SD, n = 5.

Degumming method		Degumming rate (%)	Name for silk fibres	Name for films
Boiling water	1 h	8.8 ± 0.5	SF/SS_1_	RSF/RSS_1_
	3 h	14.7 ± 0.4	SF/SS_2_	RSF/RSS_2_
	5 h	20.7 ± 0.1	SF	RSF
Na_2_CO_3_	1.5 h	22.8 ± 0.4	SF_C_	RSF_C_

### Preparation of PEG-DE-crosslinked films

PEG-DE was added to silk protein aqueous solution dropwise with stirring at a weight ratio of 1.0:0.8, then debubbled. All films were prepared by casting the same volume of mixture into polyethylene dishes (diameter 50 mm), then drying at 40 °C for 6 h while revolving slowly. Non-crosslinked films served as controls.

### Measurement of mechanical properties

The tensile properties of degummed silk fibres and films were measured using an Instron 3365 universal testing machine (Instron, Boston, MA, USA) at 20 ± 2 °C and 65 ± 2% relative humidity (RH). Degummed silk fibres were preconditioned under the above temperature and humidity conditions for 24 h before measurement. Parameters were as follows: clamp distance 250 mm, extension rate 250 mm/min, pre-tension 0.5 cN. The linear density (tex) of fibres was determined by measuring the length and mass of filaments (Eq. 1), and the specific breaking strength (N/tex) was calculated according to Eq. 2.

All films were cut into rectangles of 10 mm × 50 mm, immersed in deionised water for 1 h, and the thickness was measured by an electronic spiral micrometre. The tensile properties of films were measured in the wet state. Parameters were as follows: clamp distance 20 mm, extension rate 20 mm/min, pre-tension 0.5 cN. For each fibre or film, 10 independent samples were tested. Samples were secured into the Instron clamps and tests were run until samples failed through tearing. Breaking strength (MPa) and the breaking elongation (%) were calculated according to Eqs. (3) and (4) respectively:

1 $${\text{Linear density }}({\text{tex}}) = 1000\frac{{{\text{G}}_{{\text{K}}} }}{{{\text{L}}_{{0}} }}$$

2 $${\text{Specific breaking strength }}({\text{N}}/{\text{tex}}) = \frac{{\text{F}}}{{{\text{N}}_{{\text{t}}} }}$$

3 $${\text{Breaking strength }}({\text{MPa}}) = \frac{{\text{F}}}{{\text{S}}}$$

4 $${\text{Breaking elongation }}({\text{ \% }}) = \frac{{{\text{L}} - {\text{L}}_{0} }}{{{\text{L}}_{{0}} }}\times 100$$

where Nt (tex) is the linear density of fibres, G_k_ (g) is the quality of fibres with conventional moisture regains, F (N) is the breaking force, S (mm^2^) is the cross-sectional area of films, L (mm) is the breaking length of samples, and L_0_ (mm) is the original length of the samples.

Young’s modulus (MPa) was calculated using the secant moduli from 5 to 10% strain in the stress–strain curves.

### Structure characterisation

The chemical structure and molecular conformation of all films were analysed using a Nicolet Avatar-IR360 Fourier transform infrared spectroscopy (FTIR) instrument (Nicolet, Madison, WI, USA). Thirty-two scans were recorded with a resolution of 4 cm^−1^ and a scanning range of 500–4000 cm^−1^. In addition, we semi-quantitatively analysed the molecular conformation by deconvolution of the amide I band using Peakfit v4.12 software as reported previously^24^.

Crystalline structures of all samples were determined by a X ‘Pert-Pro MRD X-ray diffractometer (XRD; Philips, Amsterdam, The Netherlands) with a CuK α radiation source at 2θ of 5–50° and a scanning speed of 2°/min. Crystallinity was calculated through separate peak-fitting using the same quantitative analysis software as for FTIR.

### Statistical analysis

Results are presented as mean ± standard deviation (SD) of the mean. Comparison of means was performed using one-way analysis of variance (ANOVA), followed by independent Student’s *t*-tests using SPSS17.0 statistical software (IBM, Armonk, NY, USA). Statistical significance was set at *p* < 0.05.

## Results and discussion

### Tensile properties of degummed silk fibres

**Figure 1:**
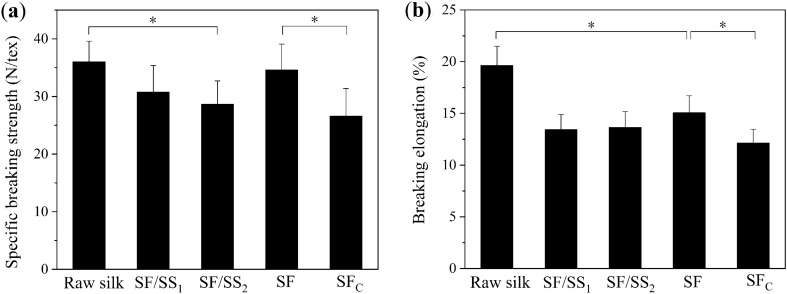
Tensile properties of degummed silk fibres with different degumming rates. (**a**) Specific breaking strength, (**b**) Breaking elongation. Results are mean ± SD, n = 10, ^*^p < 0.05.

The surface of raw silk covers a complete layer of sericin that binds two fibroin fibres tightly together^27^. Sericin can be dissolved in hot water. The degumming method or degumming degree can have a significant impact on the mechanical properties of silk fibres. Therefore, we measured the mechanical properties to evaluate the degree of damage for degummed silk fibres with different degumming rates. The degumming rate of raw silk was stable after degumming in boiling water for 5 h, which was close to that of Na_2_CO_3_ degumming. As shown in Fig. 1, the specific breaking strength and breaking elongation of raw silk decreased after degumming, especially for Na_2_CO_3_-degummed silk fibres, which decreased by 26% and 38%, respectively. Removing sericin weakened the cohesion between single fibres, hence the tensile properties of silks were significantly decreased. However, the specific breaking strength and the breaking elongation of SF fibres were significantly higher than those of SF_C_ fibres, indicating that Na_2_CO_3_ degumming caused serious damage to silk fibroin macromolecules. Due to the discontinuous distribution of residual sericin, many weak regions were generated on the surfaces of silks, which resulted in lower specific breaking strength and breaking elongation for SF/SS_1_ and SF/SS_2_ fibres compared with SF fibres.

In our preliminary experiments, repeated tests demonstrated that the Na_2_CO_3_ degumming rate was slightly higher than that of boiling water degumming for 9 h, indicating that the hierarchical structure or macromolecular chains of silk fibres were destroyed by Na_2_CO_3_ degumming, resulting in partial dissolution of silk fibroin^28^.

### Tensile properties of PEG-DE-crosslinked films

**Figure 2:**
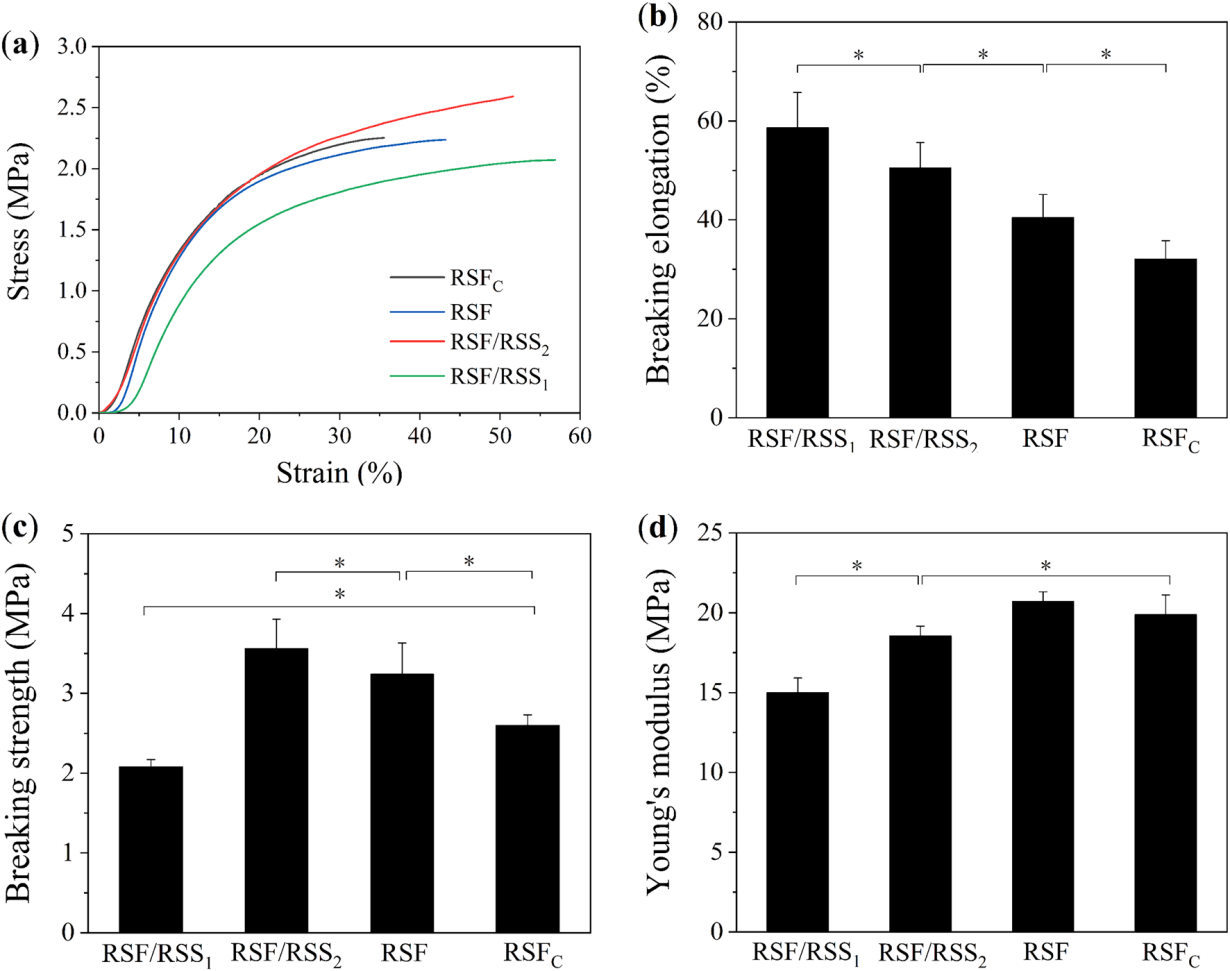
Tensile properties of PEG-DE-crosslinked silk protein films. (**a**) Stress–strain curves, (**b**) Breaking elongation, (**c**) Breaking strength, (**d**) Young’s modulus. Results are mean ± SD, n = 10, ^*^p < 0.05.

Tissue engineering materials are generally applied in a wet state, hence the effect of the degumming degree on the tensile properties of wet films was examined. Figure 2a shows the stress–strain curves of PEG-DE-crosslinked films.

The mechanical properties of regenerated silk protein-based materials depend on their molecular weights and crystallinities; the higher the molecular weight, the stronger the mechanical properties^29^. Therefore, compared with RSF_C_ film prepared by Na_2_CO_3_ degumming, the breaking strength of RSF films was increased significantly. This result is consistent with those obtained by using sodium dodecyl sulfate–polyacrylamide gel electrophoresis in our previous study; thus, boiling water degumming causes less damage to macromolecules of fibroin fibres, resulting in higher molecular weight compared with Na_2_CO_3_ degumming^24^. In the presence of sericin, the breaking elongation of RSF/RSS composite films was significantly improved, and dependent on the sericin content (Fig. 2b). Sericin macromolecules shuttled between silk fibroin macromolecular chains, and bound to silk fibroin macromolecules via hydrogen bonds or covalent bonds. Therefore, when the films were stretched, silk fibroin macromolecular chains sharply extended due to the “bridging effect” and relative slippage of the sericin macromolecules^30^. In addition, a small amount of sericin enhanced the force between silk fibroin macromolecular chains, resulting in increased breaking strength of RSF/RSS_2_ composite films. Figure 2c shows that the breaking strengths were ordered RSF/RSS_2_ film (3.56 ± 0.37 MPa) > RSF film (3.24 ± 0.39 MPa) > RSF_C_ film (2.60 ± 0.13 MPa). The breaking strengths of RSF/RSS_2_ and RSF films were equivalent to natural cornea tissue (approximately 3–5 MPa)^31^, and higher than that of RSF-based biomimetic periosteum prepared by Na_2_CO_3_ degumming^32^. Therefore, RSF film and RSF/RSS_2_ composite film were considered alternative hard-tissue repair materials such as dura or periosteum. However, when the degumming rate was lower, the higher content of sericin resulted in an increase in solution viscosity and disruption to the ordered arrangement of silk fibroin macromolecular chains^24^, leading to reduced breaking strength of RSF/RSS_1_ film to even less than that of RSF_C_ film prepared by Na_2_CO_3_ degumming.

Compared with RSF_C_ film, the Young's modulus of RSF film was increased, while that of RSF/RSS_1_ film and RSF/RSS_2_ film was decreased significantly (Fig. 2d). Notably, breaking elongation of RSF/RSS_2_ film (50.51%) was 1.6 times that of RSF_C_ film, and was significantly higher than the elongation of a propionamide/SF blend film (~ 13%) or poly(ε-caprolactone)/SF electrospun film (38.14%) used in corneal regeneration^33,34^. These results indicated that the flexibility of RSF/RSS_2_ film was markably improved, making it a candidate material for corneal tissue repair.

### Molecular conformation of PEG-DE-crosslinked films

**Figure 3:**
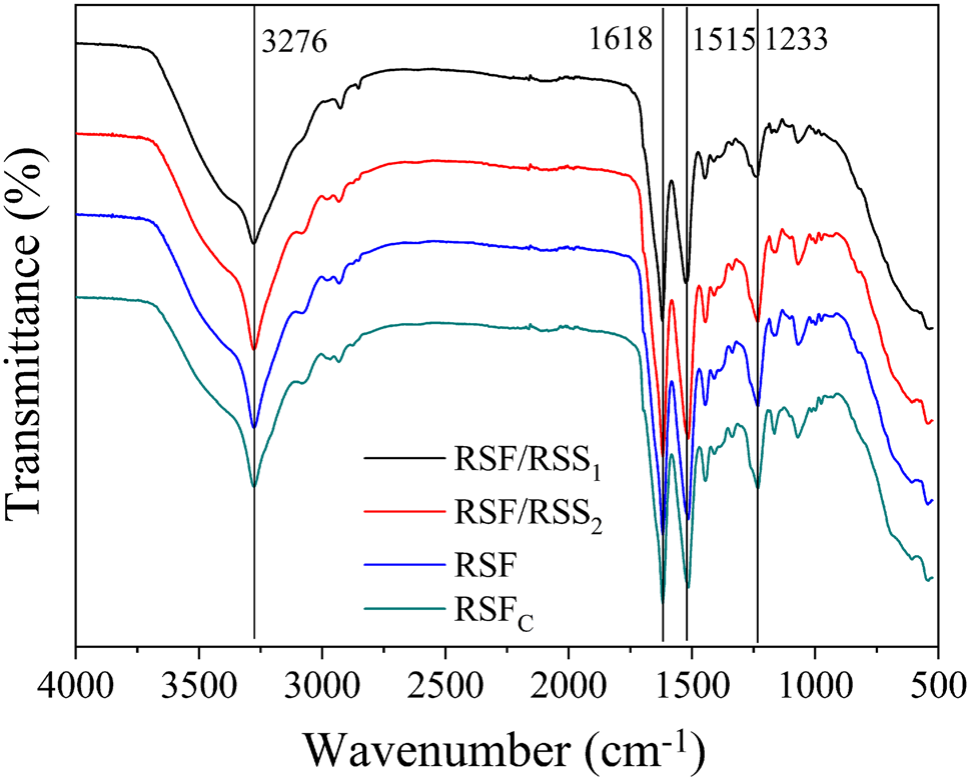
ATR-FTIR spectrum of the PEG-DE-crosslinked silk protein films.

For silk protein-based materials, the secondary structure and crystalline structure formed by self-assembly are key factors determining the mechanical properties^35^. Figure 3 shows the FTIR spectral curves of PEG-DE crosslinked films. All films showed similar characteristic bands at ~ 1618 cm^−1^ (β-sheet), 1515 cm^−1^ (β-sheet) and 1233 cm^−1^ (β-sheet), which were assigned to the C=O stretching vibration, N–H bending vibration and C–N stretching vibration, respectively^36^. Compared with non-crosslinked films^24^, the amide I peak of crosslinked films moved from 1637 cm^−1^ to 1618 cm^−1^, indicating a change from random coil to β-sheet conformation. Under the polarity effect of sericin macromolecules, PEG-DE further induced the silk fibroin macromolecular chains to extend in an orderly manner, which promoted more amide groups to form hydrogen bonds and weakened the stretching vibration of C=O. In addition, the characteristic peak near 3276 cm^−1^ became broader and stronger, indicating that more –OH groups formed new hydrogen bonds between macromolecules. Compared with RSF_C_ film, the stronger intensity at 3276 cm^−1^ for RSF/RSS composite films was ascribed to the stronger association action of –OH^37^.

Table 2 shows the molecular conformational contents of all films. In non-crosslinked films, the β-sheet content of RSF film was significantly higher than that of RSF_C_ film. This result further confirmed that boiling water degumming produced high-molecular-weight silk protein, which contributed to the formation of more stable β-sheet structure. The β-sheet content of PEG-DE-crosslinked films was obviously increased, especially in boiling water degumming groups. PEG-DE can chemically react with OH, –COOH and NH_2_ groups in silk fibroin and sericin macromolecules, which promotes crosslinking of silk protein macromolecules to form stable β-sheet structure^38^. A small amount of sericin slightly increased the β-sheet content of RSF/RSS_2_ composite film owing to an increase in functional groups. In contrast, excessive sericin in RSF/RSS_1_ composite film inhibited the orderly self-assembly of silk fibroin, resulting in relatively less β-sheet content. Thus, the β-sheet content was ordered RSF/RSS_2_ composite film > RSF film > RSF/RSS_1_ composite film, consistent with the breaking strengths of these films.

**Table 2:** Molecular conformation content of silk protein films (%). Results are means ± SD, n = 3.

Sample	β-sheet	Random coil	α-helix	β-turn
Non-crosslinking				
RSF_C_	44.5 ± 0.9	23.1 ± 0.6	9.5 ± 0.5	22.9 ± 0.7
RSF	47.3 ± 0.5	21.2 ± 0.5	8.9 ± 0.6	22.5 ± 0.4
RSF/RSS_2_	44.9 ± 0.5	25.7 ± 0.2	9.3 ± 0.3	20.1 ± 0.3
RSF/RSS_1_	44.7 ± 0.6	22.5 ± 0.3	9.5 ± 0.5	23.3 ± 0.4

PEG-DE-crosslinking				
RSF_C_	59.8 ± 0.2	14.6 ± 0.6	8.6 ± 0.2	17.0 ± 0.4
RSF	67.1 ± 0.4	9.1 ± 0.3	6.8 ± 0.6	17.1 ± 0.5
RSF/RSS_2_	67.4 ± 0.6	8.8 ± 0.6	6.6 ± 0.5	17.2 ± 0.4
RSF/RSS_1_	64.3 ± 0.7	9.6 ± 0.6	7.3 ± 0.5	18.8 ± 0.6

### Crystalline structures of PEG-DE-crosslinked films

**Figure 4:**
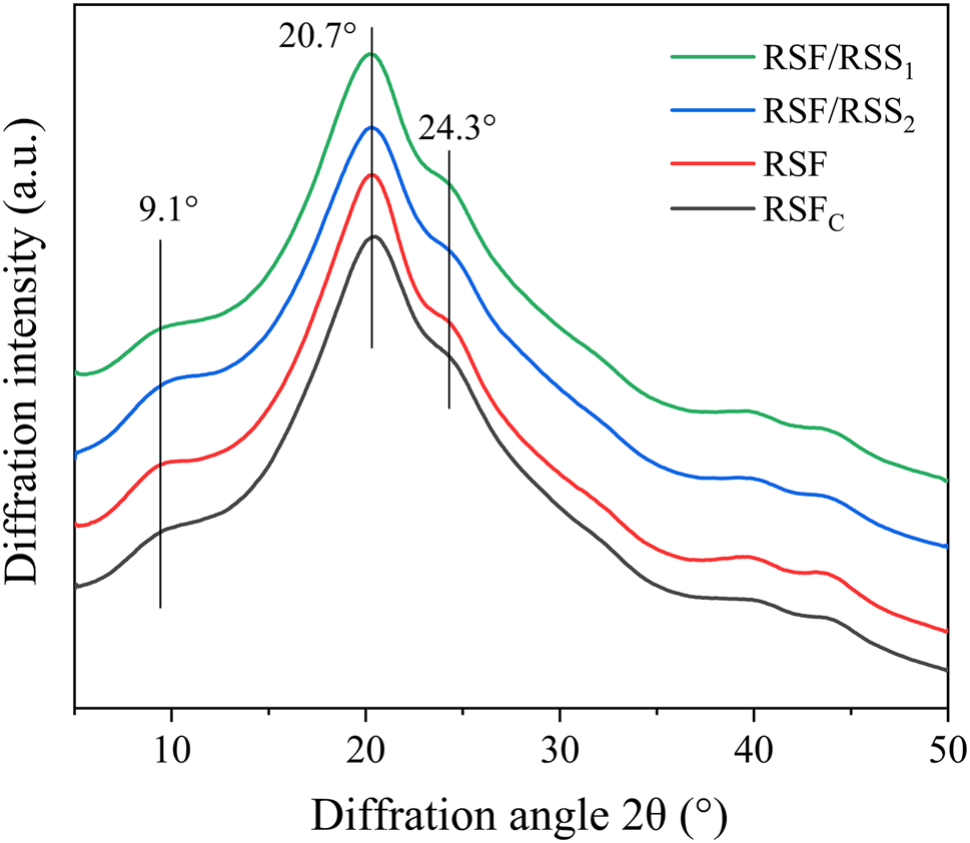
XRD patterns of the PEG-DE-crosslinked silk protein films.

The H-chain is the major protein component with a regular structural sequence and accounts for ~ 92% of the molecular weight of silk fibroin. The H-chain is composed of 12 hydrophobic regions and 11 hydrophilic regions arranged alternately^39^. The highly repetitive (AGSGAG)n sequences induce silk fibroin macromolecules to aggregate and form a dense crystalline structure^40^. Sericin, which surrounds two strands of fibroin fibres in raw silk, is an amorphous material and soluble in hot water^41^. However, RSS material can form partially crystalline aggregates induced by the hydrogen bonding effects of hydrophilic groups during the process of macromolecular self-assembly. In our previous study, there were obvious silk I crystalline diffraction peaks at 12.1° and 19.9° for non-crosslinked RSF/RSS and RSF films, and 24.7° for RSF_C_ film^24^, indicating that silk I crystals in RSF/RSS or RSF films were more abundant than in RSF_C_ film. After PEG-DE crosslinking, typical silk II crystalline peaks appeared at 9.1°, 20.7° and 24.3° for all films (Fig. 4), which indicated that PEG-DE converted silk I into silk II (β-sheets). Furthermore, the silk II crystalline peak of sericin appeared at 23.3°^42^, which enhanced the crystalline peak at 24.3° for silk fibroin, especially for RSF/RSS_1_ film. The results also confirmed that the higher the molecular weight, the more stable the aggregation structure formed under the induction of external factors.

## Conclusion

In this study, a one-step extraction method was used to prepare RSF/RSS composite solutions with uniform distribution. We developed a series of PEG-DE-crosslinked RSF/RSS composite films and RSF films with improved mechanical properties compared to those of RSF_C_ film degummed by Na_2_CO_3_. Analysis of tensile properties indicated that boiling water degumming caused less damage to fibroin fibres. Further studies showed that PEG-DE-crosslinked films prepared using boiling water degumming formed stable secondary structures and crystalline structures, and the β-sheet content was significantly higher than that of RSF_C_ film prepared by Na_2_CO_3_ degumming. Films prepared using boiling water degumming possessed significantly enhanced breaking strength and flexibility. The study provided new strategies for application of silk protein in dura, periosteum and corneal tissue repair. In addition, the full utilisation of sericin and the absence of chemicals in the degumming process were of benefit to environmental protection.

## Data Availability

The datasets used and/or analysed during the current study available from the corresponding author on reasonable request.
